# Prevalence and risk factors for age-related macular degeneration in a population-based cohort study of older adults in Northern Ireland using multimodal imaging: NICOLA Study

**DOI:** 10.1136/bjo-2021-320469

**Published:** 2022-10-10

**Authors:** Ruth E Hogg, David M Wright, Nicola B Quinn, Katherine Alyson Muldrew, Barbra Hamill, Laura Smyth, Amy Jayne McKnight, Jayne Woodside, Mark A Tully, Sharon Cruise, Bernadette McGuinness, Ian S Young, Frank Kee, Tunde Peto, Usha Chakravarthy

**Affiliations:** 1 Centre for Public Health, Queen's University Belfast Faculty of Medicine Health and Life Sciences, Belfast, UK; 2 Ulster University, Newtownabbey, UK; 3 Belfast Faculty of Engineering and Physical Sciences, Queen's University, Belfast, UK; 4 Centre for Public Health, Queen's University Belfast Faculty of Arts Humanities and Social Sciences, Belfast, UK

**Keywords:** epidemiology, genetics, imaging, macula, retina

## Abstract

**Purpose:**

To report prevalence and risk factor associations for age-related macular degeneration (AMD) and AMD features from multimodal retinal grading in a multidisciplinary longitudinal population-based study of aging in Northern Ireland.

**Study design:**

Population-based longitudinal cohort study.

**Methods:**

Retinal imaging at the Norther Ireland Cohort for the Longitudinal Aging Study health assessment included stereo Colour Fundus Photography (CFP) (Canon CX-1, Tokyo, Japan) and Spectral-Domain Optical Coherence Tomography (SD-OCT) ((Heidelberg Retinal Angopgraph (HRA)+OCT; Heidelberg Engineering, Heidelberg, Germany). Medical history and demographic information was obtained during a home interview. Descriptive statistics were used to describe the prevalence of AMD and individual AMD features. Multiple imputation followed by multiple regression modelling was used to explore risk factor associations including relationships with AMD genetic risk score.

**Results:**

Retinal images from 3386 participants were available for analysis. Mean age of the sample was 63.4 (SD 9.01, range: 36–99). Population weighted prevalence of AMD using colour grading in those over 55 years was: no drusen: 6 0.4%; drusen <63 μm: 15.9%; drusen 63–125 µm: 13.7%; drusen >125 µm or pigmentary changes: 8.3%; late AMD: 1.6%. Prevalence of AMD features in those over 55 years was: OCT drusen 27.5%, complete outer retinal pigment epithelium and outer retinal atrophy (cRORA) on OCT was 4.3%, reticular drusen 3.2% and subretinal drusenoid deposits 25.7%. The genetic risk score was significantly associated with drusen and cRORA but less so for SDD alone and non-significant for hyperpigmentation or vitelliform lesions.

**Conclusions:**

Multimodal imaging-based classification has provided evidence of some divergence of genetic risk associations between classical drusen and SDD. Our findings support an urgent review of current AMD severity classification systems.

**WHAT IS ALREADY KNOWN ON THIS TOPIC**

- Age-related macular degeneration (AMD) is of significant public health concern due to the impact of vision loss on quality of life together with an increasingly aged population. Substantial advances in retinal imaging has furthered our understanding of the condition through the use of optical coherence tomography (OCT) images, yet most epidemiological studies rely solely on colour fundus photographs for assessment.

**WHAT THIS STUDY ADDS**

- The Norther Ireland Cohort for the Longitudinal Aging Study included multimodal retinal imaging (colour fundus photography, OCT and ultrawide field retinal imaging enabling an unprecedented assessment of AMD including individual retinal features of the condition. The results show disparities between assessments using different imaging modalities highlighting the importance of using multimodal imaging for future studies of prevalence and incidence.

**HOW THIS STUDY MIGHT AFFECT RESEARCH, PRACTICE OR POLICY**

- This study supports the incorporation of OCT based features into future grading schemes or severity staging systems and the need for longitudinal data on progression of OCT based features to better understand their significance in progression to late-stage disease.

## Introduction

Epidemiological studies of age-related macular degeneration (AMD) have traditionally relied on colour fundus photography (CFP) to identify the characteristics used to classify each eye into disease severity stages. Landmark studies such as the Beaver Dam Eye study,^1^ the Rotterdam Eye Study^2^ and the Blue Mountain’s Eye Study^3^ have identified the key phenotypic features and revealed the natural history of progression and associations with risk factors especially age, smoking and genetic risk.^4–7^ Although studies showed the importance of drusen size and hyperpigmentation in influencing the risk of progression, the role of other colour-defined features such as reticular pseudodrusen^8^ (RPD; a characteristic form of extracellular drusenoid deposits) and small drusen remains controversial. This is mainly because colour grading can overestimate presence of small drusen^9^ and underestimate presence of RPD.^10^ Optical Coherence tomography (OCT permits the distinction of nodular (classic) drusen from RPD through characterisation of layer location.

The establishment of a new population-based longitudinal study of ageing in Northern Ireland (The Northern Ireland Cohort for the Longitudinal Study of Aging (NICOLA)) offered an opportunity to assess the retina using multimodal retinal imaging (CFP, OCT, ultrawide field imaging (UWFII)) approach, define previously unexplored phenotypes from an epidemiological perspective and study risk factor associations with an extended range of markers of systemic health.

## Methods

### NICOLA Study overview

**Figure 1:**
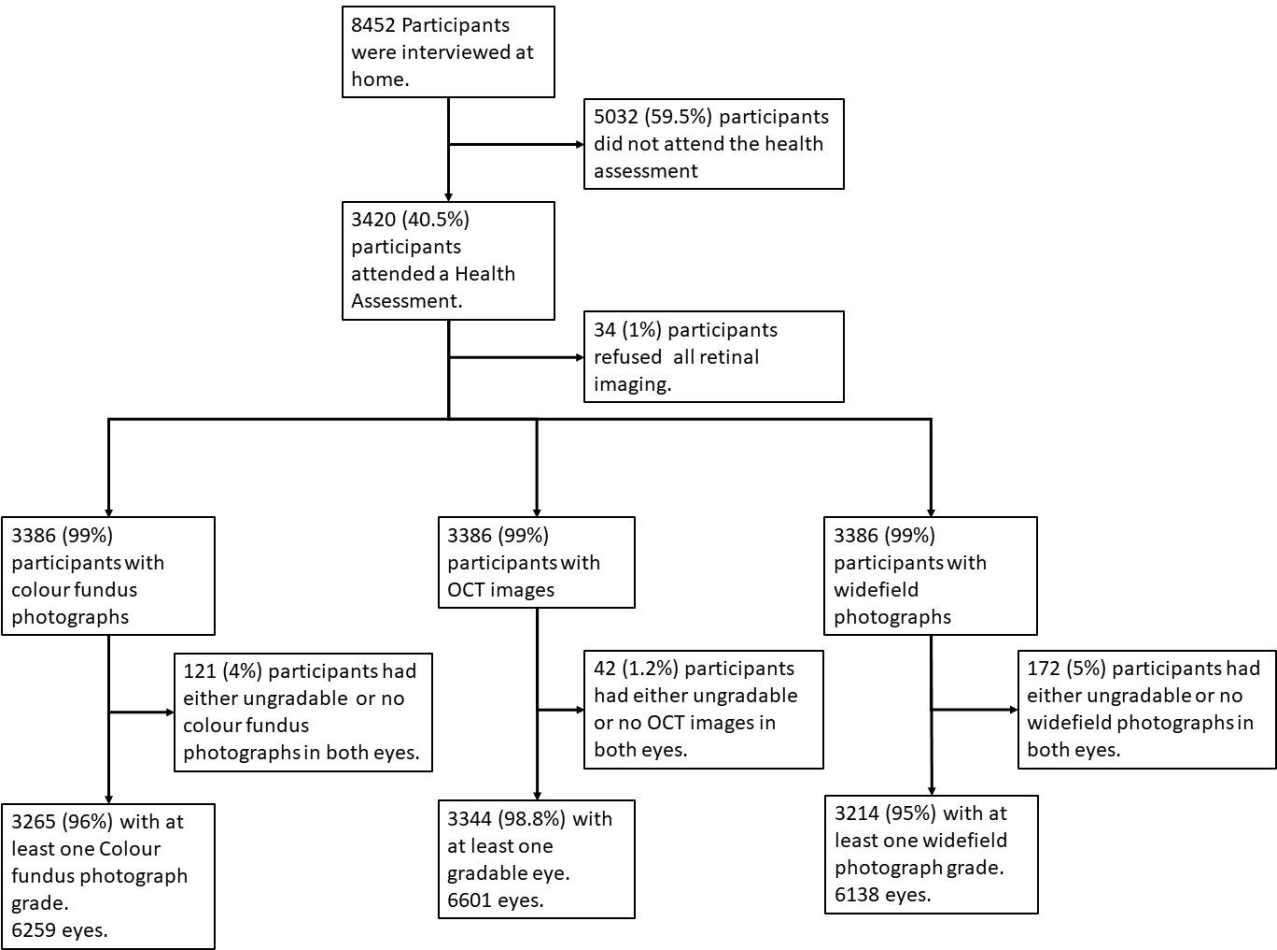
Flow chart showing participant pathway in wave 1 of the NICOLA Study. NICOLA, Northern Ireland Cohort for the Longitudinal Study of Aging.

The NICOLA is a multidisciplinary prospective population-based cohort study. Wave 1 of the study commenced in December 2013 and ended in April 2018. In total, 8452 persons completed the home-based computer-assisted personal interview. Of these, 3420 (40.5%) attended for the health assessment, which consisted of anthropometric, cardiac, respiratory, cognitive and ophthalmic tests (figure 1).

Details on image acquisition, image grading, physical data collection, self-report, blood-based biomarkers, genetic analysis, outcome categorisation and covariate selection can be found in the online supplemental methods.

10.1136/bjo-2021-320469.supp1 Supplementary data

### Statistical analysis

All statistical analysis was conducted using R (V.3.6.0).^11^

### Prevalence analysis

Prevalence of AMD was estimated, weighting records by the population distribution of age and sex as recorded in the 2011 Census for Northern Ireland. For the eye level analysis, the frequency, prevalence and 95% CIs for each AMD feature was calculated. To facilitate comparison with prior studies,^12^ estimates are provided by age category and with a separate estimate for participants aged 55 years and above.

### Imputation and risk factor association

Missing risk factor values were imputed using multivariate imputation by chained equations (MICE) using the *R* package *mice* (V.3.8.0),^13^ which yielded a set of five datasets in which the missing values had been imputed probabilistically. The regression models outlined further were fitted using each of the imputed datasets, and estimates were pooled across imputations. A single estimate was generated for each parameter of interest that encompassed the additional uncertainty introduced by imputation. Further details of the imputation procedure are given in online supplemental methods.

Associations between AMD features and risk factors were explored using logistic regression. A series of regressions were fitted for each AMD feature with the feature as the response variable. First, a set of univariate models was fitted, the sole predictor in each being one of the candidate risk factors. A second set was adjusted with the addition of age (linear and quadratic terms) and sex. Risk factors receiving some statistical support in the age-adjusted andsex-adjusted models (p<0.1) were included in a multivariable model. Finally, a saturated model including all risk factors was fitted. Where an AMD feature was particularly rare, inclusion of all the selected variables occasionally resulted in unstable estimates or a failure of multivariable or saturated models to converge. In these instances, problematic combinations of predictors were identified, and models were refitted after removing these risk factors. For the analysis of person-level AMD risk factors (dichotomised AMD stage), an additional model was fitted excluding the Genetic Risk Score (GRS) to explore the extent to which associations with other risk factors differed in the absence of the GRS.

## Results

### Cohort characteristics

Of the 3420 participants who attended the health assessment, retinal imaging was performed in 3386 (99%) (figure 1). Images were available for colour 6259 eyes (92%), OCT 6601 (96%) and UWFI 6138 (89%). Images were ungradable in approximately 121 (4%) (colour), 42 (1%) (OCT) and 172 (5%) (UWFI) eyes. The number of individuals for whom both eyes were gradable was: colour 2994 (88%), OCT 3257 (95%) and UWFI 2934 (85%). More than 85% of participants had gradable images from both eye on all three imaging modalities. Online supplemental table 1 shows proportions of participants with ungradable images by imaging modality. The mean (SD) age of participants with gradable colour images was 63.5 (8.9) years (online supplemental table 1), and over 99.9% were Caucasian.

10.1136/bjo-2021-320469.supp2 Supplementary data

### Prevalence by Beckman stage

The prevalence of stage 0 and stage 1 (representing no features of AMD or small hard drusen only) declined from 62.7% and 23.7% to 54.3 % and 10.9%, respectively (table 1). For stage 2, the prevalence rose steadily from 9.6% to 17.3% in those in age band 75–84 but was 7.1% in the oldest age band. For participants in stage 3, the prevalence increased from 4.0% to 16.9%. Stage 4 representing the most advanced cases of AMD was rare in the two younger age bands, was infrequent in the age groups between 65 and 84 and occurred at 10.9% in the oldest age group. Prevalence rates were similar across sexes (online supplemental table 2).

**Table 1:** Prevalence by stage of Beckman clinical classification for AMD % (95% CI) by age

Unweighted					Weighted						
Age (years)	Number at risk	0	1	2	3	4	0	1	2	3	4
All ≥55	2690	61.1 (59.2 to 63.0)	16.6 (15.2 to 18.1)	13.8 (12.5 to 15.1)	7.5 (6.6 to 8.6)	1.0 (0.6 to 1.4)	60.4 (58.3 to 62.6)	15.9 (14.5 to 17.5)	13.7 (12.3 to 15.2)	8.3 (7.1 to 9.8)	1.6 (0.9 to 2.6)
<55	575	63.1 (59.0 to 67.1)	23.7 (20.2 to 27.3)	9.4 (7.1 to 12.1)	3.8 (2.4 to 5.7)	0.0	62.7 (58.5 to 66.7)	23.7 (20.2 to 27.5)	9.6 (7.3 to 12.4)	4.0 (2.5 to 6.0)	0.0
55–64	1257	64.6 (61.9 to 67.2)	18.5 (16.3 to 20.7)	12.2 (10.4 to 14.1)	4.7 (3.6 to 6.0)	0.1 (0.0 to 0.4)	64.5 (61.8 to 67.2)	18.6 (16.4 to 20.8)	12.1 (10.3 to 14.0)	4.8 (3.7 to 6.1)	0.1 (0.0 to 0.4)
65–74	1061	59.3 (56.3 to 62.3)	16.0 (13.9 to 18.4)	14.9 (12.8 to 17.2)	8.8 (7.1 to 10.6)	1.0 (0.5 to 1.8)	59.0 (55.9 to 62.0)	16.1 (13.9 to 18.4)	15.2 (13.1 to 17.5)	8.7 (7.1 to 10.6)	1.0 (0.5 to 1.8)
75–84	325	54.8 (49.2 to 60.3)	12.3 (8.9 to 16.4)	16.9 (13.0 to 21.5)	13.2 (9.7 to 17.4)	2.8 (1.3 to 5.2)	55.6 (49.9 to 61.2)	11.3 (8.1 to 15.2)	17.3 (13.2 to 22.0)	13.0 (9.5 to 17.2)	2.8 (1.2 to 5.3)
≥85	47	53.2 (38.1 to 67.9)	10.6 (3.5 to 23.1)	8.5 (2.4 to 20.4)	17.0 (7.6 to 30.8)	10.6 (3.5 to 23.1)	54.3 (36.7 to 71.1)	10.9 (2.9 to 26.1)	7.1 (1.4 to 19.9)	16.9 (6.4 to 33.3)	10.9 (2.9 to 26.1)

Table 1 shows the prevalence of AMD severity stages based on the Beckman clinical classification using the worse eye (person level). Unweighted and weighted estimates are shown. Data were weighted by age and gender using the 2011 Census for Northern Ireland.

AMD, age-related macular degeneration.

### Prevalence by graded features

The prevalence of features of AMD based on colour or OCT grading is shown in table 2.

**Table 2:** Prevalence of AMD features by age category (eye-level analysis, unweighted)

Colour/*en face* grading							Multimodal grading (Infra-red and OCT)						
Age (years)	Number at risk	Hyperpigmentation	Drusen (any size)	Drusen ≥63 µm	Drusen ≥125 µm	Reticular Drusen	GA	Number at risk	Drusen (any size)	SDD	cRORA	CNV	Vitelliform
All ≥55	5128	2.0 (1.5, 2.4)	28.9 (27.4 to 30.4)	14.5 (13.4 to 15.7)	3.9 (3.3 to 4.5)	3.2 (2.7 to 3.9)	0.3 (0.2 to 0.6)	5450	27.5 (26.1 to 29.0)	25.7 (24.3 to 27.1)	4.3 (3.7 to 5.0)	0.3 (0.2 to 0.5)	0.3 (0.2, 0.6)
<55	1131	1.0 (0.5, 1.7)	26.0 (23.0 to 29.2)	7.9 (6.1 to 9.9)	1.3 (0.7 to 2.4)	1.3 (0.6 to 2.5)	0.0	1151	16.3 (13.9 to 19.0)	13.0 (10.9 to 15.4)	2.5 (1.5 to 3.9)	0.0	0.0
55–64	2447	1.0 (0.6, 1.6)	24.7 (22.7 to 26.8)	10.2 (8.8 to 11.6)	1.9 (1.4 to 2.6)	1.1 (0.7 to 1.7)	0.0	2534	20.4 (18.5 to 22.3)	18.3 (16.6 to 20.1)	2.9 (2.2 to 3.7)	0.0 (0.0 to 0.2)	0.0 (0.0, 0.2)
65–74	2003	2.4 (1.7, 3.3)	30.6 (28.2 to 33.1)	16.4 (14.5 to 18.5)	4.7 (3.6 to 6.0)	3.8 (2.8 to 5.1)	0.5 (0.2 to 1.1)	2144	29.5 (27.1 to 31.9)	27.3 (25.1 to 29.6)	4.7 (3.7 to 5.8)	0.2 (0.1 to 0.7)	0.6 (0.2, 1.1)
75–84	595	3.2 (1.8, 5.2)	38.8 (33.9 to 43.9)	24.7 (20.5 to 29.4)	8.2 (5.7 to 11.4)	8.6 (5.9 to 11.9)	1.2 (0.4 to 2.6)	675	44.3 (39.5 to 49.2)	44.1 (39.5 to 48.9)	6.8 (4.7 to 9.5)	0.8 (0.3 to 1.9)	0.6 (0.1, 2.1)
≥85	83	9.6 (3.4, 20.5)	39.8 (26.8 to 53.9)	22.9 (12.1 to 37.1)	9.6 (3.4 to 20.5)	12.0 (4.0 to 26.0)	0.0	97	54.6 (40.9 to 67.9)	53.6 (40.1 to 66.8)	16.5 (8.2 to 28.2)	6.0 (2.0 to 13.5)	2.1 (0.0, 11.1)

Table 2 shows the prevalence of AMD features on en face and multimodal grading (non-weighted) (eye level). The all category is restricted to those over 55 years to enable comparison with other studies who restrict definition of AMD to this age category.

AMD, age-related macular degeneration; CNV, choroidal neovascular membrane; cRORA, complete retinal pigment epithelium and outer retinal atrophy; GA, geographic atrophy; OCT, optical coherence tomography; SDD, subretinal drusenoid deposit.

With colour detection, the prevalence of focal hyperpigmentation increased from 1.0% to 9.6% with age. Small drusen <63 µm rose from 7.9% in the youngest to 22.9% in those older than 85 years. The prevalence of drusen >125 µm rose steadily from 1.3% to 9.6% doubling by each age category, but the oldest two age bands were similar. RPD frequencies were similar for the two youngest age bands but showed steady rises in the three oldest age categories. On OCT images, subretinal drusenoid deposit (SDD) were observed frequently on OCT in all age bands compared with RPD on colour. These differentials were striking 18.0% on OCT versus 1.1% on colour in the youngest age category and 53.6% versus 12.0% in those >85 years. The prevalence of geographic atrophy (GA) was low on colour and only seen in the two oldest age bands at 0.5% and 1.2%. This contrasted with the higher proportions of cRORA on OCT, which rose from 2.5% from those under 55 year to 16.5% in those aged 85 years and above. The prevalence of all the graded features was not significantly different between men and women (online supplemental table 3).

### Prevalence of AMD features on UWFI colour imaging

The prevalence of hard and soft drusen and hyperpigmentation in UWFI colour images by age groups are shown in table 3.

**Table 3:** Prevalence of hard drusen, soft drusen and hyperpigmentation on ultrawide field retinal images (weighted)

Age (years)	Number at risk	Centre	Mid	Far	Full
Hard drusen					
All ≥55	5023	62.8 (61.0 to 64.7)	86.8 (85.4 to 88.1)	75.6 (74.0 to 77.2)	94.6 (93.7 to 95.4)
<55	1115	58.6 (55.4 to 61.7)	85.2 (82.7 to 87.4)	76.9 (74.1 to 79.6)	94.4 (92.6 to 95.8)
55–64	2392	57.0 (54.8 to 59.2)	86.4 (84.8 to 87.9)	77.2 (75.2 to 79.0)	94.9 (93.8 to 95.8)
65–74	1971	64.6 (62.2 to 67.0)	87.8 (86.2 to 89.3)	77.0 (74.8 to 79.0)	94.7 (93.6 to 95.7)
75–84	585	69.8 (65.2 to 74.0)	88.2 (84.9 to 91.0)	72.6 (68.2 to 76.6)	94.9 (92.8 to 96.6)
≥85	75	70.8 (52.3 to 85.2)	80.4 (64.3 to 91.4)	68.2 (52.7 to 81.2)	91.2 (80.3 to 97.2)
Soft Drusen					
All≥55	5023	5.6 (4.6 to 6.9)	3.2 (2.6 to 3.9)	3.7 (3.1 to 4.5)	8.6 (7.4 to 10.0)
<55	1115	0.8 (0.3 to 1.7)	0.6 (0.2 to 1.3)	2.2 (1.2 to 3.5)	2.8 (1.7 to 4.2)
55–64	2392	2.3 (1.7 to 3.1)	2.1 (1.5 to 2.8)	3.4 (2.5 to 4.4)	5.5 (4.4 to 6.7)
65–74	1971	6.5 (5.2 to 8.0)	4.3 (3.2 to 5.5)	4.7 (3.6 to 6.0)	10.0 (8.4 to 11.8)
75–84	585	10.0 (7.1 to 13.5)	4.5 (2.6 to 7.1)	4.2 (2.4 to 7.0)	12.8 (9.6 to 16.7)
≥85	75	10.2 (1.9 to 27.9)	1.3 (0.1 to 4.9)	0.0	10.2 (1.9 to 27.9)
Hyperpigmentation					
All≥55	5023	2.0 (1.4 to 2.9)	8.5 (7.1 to 10.2)	12.5 (10.9 to 14.2)	14.5 (12.8 to 16.4)
<55	1115	0.8 (0.3 to 1.7)	2.1 (1.2 to 3.3)	3.2 (2.1 to 4.6)	4.6 (3.2 to 6.2)
55–64	2392	0.9 (0.5 to 1.5)	3.9 (3.0 to 4.9)	6.1 (5.0 to 7.4)	7.6 (6.3 to 9.0)
65–74	1971	1.9 (1.3 to 2.7)	8.9 (7.4 to 10.5)	13.5 (11.7 to 15.5)	15.5 (13.5 to 17.6)
75–84	585	3.1 (1.6 to 5.6)	11.0 (8.0 to 14.6)	19.0 (15.2 to 23.4)	20.9 (16.8 to 25.4)
≥85	75	6.8 (1.5 to 18.4)	27.9 (13.4 to 46.8)	27.8 (13.3 to 46.9)	34.7 (17.9 to 54.7)

Table 3 shows the prevalence of hard, soft drusen and hyperpigmentation on ultrawide field retinal images (weighted) (eye level). Unweighted and weighted estimates are shown. Data were weighted by age and gender using the 2011 Census for Northern Ireland. The all category is restricted to those over 55 years to enable comparison with other studies who restrict definition of AMD to this age category.

Small hard drusen were highly prevalent in the central (62.8%), mid (86.8%) and far periphery (75.8%) of the fundus with the highest prevalences seen in the midregion, but no significant differences were seen with age and sex (sex data not shown). The prevalence of soft drusen rose monotonically with age in the central fundus, with similar rises in the mid and far periphery. Hyperpigmentation increased with age in all three regions, but the prevalence by age group and rise with increasing age group was most marked in the far periphery. Table 4 shows the relationship between Beckman stage and the UWFI grades. The UWFI-based prevalence of soft drusen in central, mid and far regions rose with increasing Beckman stage.

**Table 4:** Comparison of Beckman stages graded on colour with presence of hard drusen, soft drusen or hyperpigmentation on UWF

	Beckman classification	Number at risk	Centre % (95% CI)	Mid % (95% CI)	Far % (95% CI)
Hard drusen	0	4131	58.4 (56.8 to 60.0)	86.0 (84.9 to 87.2)	75.8 (74.3 to 77.2)
	1	836	67.5 (64.0 to 70.8)	88.2 (85.7 to 90.3)	78.1 (75.1 to 80.9)
	2	545	68.6 (64.4 to 72.6)	90.3 (87.2 to 92.9)	85.0 (81.5 to 88.0)
	3	277	69.7 (63.9 to 75.1)	88.1 (83.4 to 91.8)	79.1 (73.0 to 84.3)
	4	31	54.8 (32.8 to 75.5)	71.0 (50.3 to 86.8)	67.7 (48.0 to 83.7)
	Ungradable	318	57.2 (51.5 to 62.8)	83.3 (78.7 to 87.3)	63.2 (57.1 to 69.0)
Soft drusen	0	4131	1.5 (1.1 to 1.9)	1.9 (1.5 to 2.5)	2.9 (2.3 to 3.5)
	1	836	2.9 (1.8 to 4.4)	2.8 (1.8 to 4.1)	4.7 (3.2 to 6.5)
	2	545	11.6 (8.7 to 15.0)	5.3 (3.5 to 7.8)	7.0 (4.8 to 9.8)
	3	277	28.5 (22.3 to 35.4)	6.5 (3.7 to 10.4)	4.3 (1.9 to 8.3)
	4	31	38.7 (18.8 to 61.9)	25.8 (9.3 to 49.7)	25.8 (9.3 to 49.7)
	Ungradable	318	6.3 (3.7 to 9.9)	2.5 (1.0 to 5.3)	1.9 (0.7 to 4.1)
Hyperpigmentation	0	4131	1.0 (0.7 to 1.4)	5.5 (4.7 to 6.4)	9.0 (8.0 to 10.1)
	1	836	0.6 (0.2 to 1.4)	5.9 (4.3 to 7.8)	8.0 (6.1 to 10.3)
	2	545	2.0 (0.9 to 3.8)	9.0 (6.4 to 12.2)	12.3 (9.3 to 15.8)
	3	277	9.0 (5.7 to 13.5)	9.0 (5.6 to 13.5)	13.7 (9.6 to 18.8)
	4	31	16.1 (5.3 to 34.1)	16.1 (5.0 to 35.0)	19.4 (6.9 to 38.9)
	Ungradable	318	0.9 (0.2 to 2.7)	5.7 (3.3 to 9.0)	9.4 (6.3 to 13.4)

UWF, ultrawide field.

The prevalence of hard drusen in all UWFI regions was similar across all Beckman stages. The prevalence of hyperpigmentation increased with increasing Beckman stage.

### Genetic risk score (GRS)

The GRS ranged from −3.0 to 5.1 (SD 1.1) and was normally distributed. In controls (Beckman 0,1), the mean GRS was 0.4 (SD 1.1). For cases (Beckman 2,3,4), the GRS was higher 0.7 (SD 1.2) (online supplemental table 4). Mean GRS (SD) for AMD features detected on colour were hyperpigmentation: 0.4 (1.2), drusen (any size): 0.6 (1.2), drusen ≥63 µm: 0.7 (1.2), drusen >125 µm: 1.0 (1.4), reticular drusen: 1.0 (1.2) and geographic atrophy: 1.6 (0.8). The mean GRS for AMD features detected on OCT were classic drusen 0.8 (1.2), SDD 0.7 (1.2), SDD only 0.5 (1.1), cRORA 0.8 (1.3), CNV 1.4 (1.1) and vitelliform lesion 0.1 (1.1).

### Risk factor associations

Comparison between stage 0 or 1 versus stages 2, 3 and 4 (online supplemental table 4) showed that only the GRS (OR 1.25, 95% CI 1.15 to 1.35, p<0.001) and serumHigh Density Lipoprotein (HDL) remained highly statistically significant in the fully adjusted model. Self-reported chronic lung disease (OR 1.71, 95% CI 1.11 to 2.64, p=0.014), Parkinson’s disease (OR 5.10, 95% CI 1.39 to 18.74, p=0.014) and the category of elevated hypertension (OR 1.44, 95% CI 1.06 to 1.96, p=0.020) just reached significance in the fully adjusted model. The association with high-sensitivity C-Reactive Protein (hsCRP) was higher in the model with GRS removed (OR 1.09, 95% CI 1.00 to 1.19, p=0.045) than when it was included (OR 1.08, 95% CI 0.99 to 1.18, p=0.065).

Risk factor associations with individual features detected by colour grading are shown in online supplemental table 5. The GRS was consistently associated with classical drusen and RPD. The OR for classical drusen <63 µm was 1.24 (1.13 to 1.37) and increased to 1.52 (1.27 to 1.81) for drusen >125 µm. The OR for RPD was 1.47 (1.23 to 1.75). There was no significant association for GRS and hyperpigmentation. The presence of a thickened choroid was significantly associated with drusen of any size.

Online supplemental table 6 shows the risk factor associations with individual grading features based on OCT. A consistent and highly significant association was seen between the GRS and classical drusen with an OR of 1.33 (95% CI 1.24 to 1.43, p<0.001). Eyes with SDD with classical drusen also had a statistically significant association OR 1.35 (95% CI 1.26 to 1.44, p<0.001). For eyes with SDD without classical drusen, the association with the GRS lost significance 1.15 (95% CI 1.02 to 1.28, p=0.017), but the direction of risk remained unchanged. The OR for cRORA was statistically significant p=0.003 at 1.26 (95% CI 1.08 to 1.46). Classical drusen were significantly associated with thick choroid OR 1.84 (95% CI 1.42 to 2.37, p<0.001) and RPD with thin choroid with an OR of 1.69 (95% CI 1.27 to 2.23, p<0.001).

## Discussion

NICOLA Study ascertained the prevalence of features of early AMD through traditional colour imaging and extended this to include UWFI and SD-OCT, permitting us to distinguish between participants based on individual characteristics. We showed clear and steady almost monotonic age-related rises in the prevalence of Beckman stages 2 and 3 representing early and intermediate AMD, respectively (table 1). These findings are in accord with prior epidemiological studies.^2 3^

In the eye- level analysis, differences by age group in the frequencies of drusen were seen on comparing features detected by colour versus OCT. Notably, on colour, the prevalence of drusen (any size) was higher than that seen on OCT in the younger age groups, and although it rose with age, the increase was shallow. On OCT, the prevalence of any drusen, which was around 16% in the youngest age band, rose steadily to 54% in the oldest age band. These colour versus OCT discrepancies mainly occurred in the detection of drusen <63 µM. Our data suggest that small drusen were more likely to be graded as present in younger age groups and likely represented an over calling of this feature by the graders.^9 14^ By contrast, small drusen when present were more likely to be missed on colour grading in the older age groups in whom there is a higher prevalence of lens opacities with resultant degradation of image quality interfering with the detection process.

We observed good correspondence for prevalence of drusen >125 µM on comparing detection by colour or by OCT. We demonstrated poor agreement for the prevalence RPD comparing detecting by *en face* technologies versus its OCT correlate of SDD, a finding that is in keeping with previous studies.^10 15 16^ We expected that the OCT correlate of pseudo drusen, SDDs would be detected at higher frequency than that reported by *en face* imaging, but the magnitude of the difference was surprising. Even though the prevalence on en face imaging of RPD was marginally lower in NICOLA Study at 3.2% than the Rotterdam study (4.9%) or that reported from another UK cohort (5.06%),^17^ it was still much higher than that of a large community-based cohort study in Australia^18^ (0.41%). Studies that have either used OCT alone or in conjunction with other imaging modalities have generally reported high prevalence rates of SDD. Alienor found the prevalence of SDD to be 13.4% using multimodal imaging^19^ in contrast to the Alstar study who reported a prevalence of 32% in their clinic based enrolment cohort^20^ using multimodal imaging. In NICOLA Study, we found the prevalence to be 25.7% in those aged 55 years and older. We contend that NICOLA Study offers better representation of the true population prevalence of SDD in older adults as its community-based sampling strategy is less likely to be biased in the direction of persons with other ocular morbidities that are common in clinic-based samples.

As with other epidemiological studies, the prevalence of large areas of atrophy visible on colour images representing GA was low, precluding generation of robust estimates of prevalence and risk factor associations for this late stage of AMD. Nonetheless, we were able to characterise in detail the presence of cRORA, an OCT based definition of focal atrophy in the outer retina,^21^ using SD-OCT, and indeed NICOLA is the first epidemiological study to record its prevalence. The proportion of eyes exhibiting cRORA even in the younger age groups was around 2.5%, and this rose steadily with age allowing us to provide robust estimates by age band for focal outer retinal atrophy. This information will be particularly useful for sample size calculations when developing protocols for GA interventional trials. In this context, we recognise that while it is presently unknown if cRORA is a robust precursor of geographic atrophy, longitudinal case series^22^ and data from clinical trials^23^ that have enrolled participants with early AMD who have progressed to GA strongly support this view.

Peripheral retinal changes have been reported in several clinical AMD cohorts^24 25^ and one other population-based study.^26^ The prevalence in the periphery of small hard drusen, soft drusen and pigmentary irregularities in the UWFI images in the NICOLA population were in accord with the high rates of abnormalities reported in these prior studies. It was notable that increasing Beckman severity stages was mirrored by increases in soft drusen and hyperpigmentation in the central, mid and far periphery. By contrast, the prevalence of hard drusen was similar in the central mid and far periphery across all Beckman severity stages suggesting that these are a ubiquitous finding. Histological studies however dispute these findings and show that the pathology visible in the periphery is not the same as that in the macula.^27^ Widefield OCT images should be prioritised in future studies as these may help resolve these disparities in clinical cohorts.

Various risk factors for intermediate and late AMD have been identified in longitudinal epidemiological studies^28^ and clinical cohorts.^29 30^ The fully adjusted multivariate regression model revealed that age and the GRS were the only highly statistically significant associations with the Beckman severity stage person-level classification. Associations between AMD and chronic lung disease is relatively novel though has recently been reported from a populatio-based retrospective study in Taiwan^31^ so deserves further exploration. Some factors such as physical activity that were significant in univariate are likely to be highly collinear with age, hence the drop from age-adjusted models. The ORs and risk estimates for the GRS and Beckman stages of intermediate and late AMD are in accord those of the eye-risk consortium, which recently reported a similar mean GRS from their large, pooled analysis of cross-sectional data from the European Eye Epidemiology Consortium.^32^

In the AMD feature-level analysis, the GRS was strongly associated with classical drusen, on separating eyes with SDD from eyes with both SDD and classical drusen, the association with the GRS lost significance despite previous studies showing a significant association between SDD and the two major AMD risk loci independent of drusen presence (ARMS2 positively associated and CFH Y402H negatively associated).^33^ We also observed that GRS was not significantly associated with hyperpigmentation when this feature present in the absence of drusen. This is not surprising since focal hyperpigmentation on its own can represent pathology such as past inflammation.

All types of drusen were associated with a thicker choroid, which is in keeping with many previous studies,^34–36^ whereas those with SDD alone had a significantly thinner choroid. A detailed study of the relationship between choroidal thickness, choroidal vascularity index (CVI) and SDD presence by Keenan *et al* 35 reported a biphasic alteration in choroidal dimensions across the disease spectrum with those with large drusen showing increased choroidal thickness and increased CVI, whereas the same parameters in those with advanced AMD in the fellow eye were no different to controls. Those with just SDD had significantly thinner choroid and reduced CVI.^35^ Keenan *et al* propose CVI as a potential biomarker of ageing given its significant and negative correlation with age, and our data on SDD alone suggest that it too may be more reflective of ubiquitous ageing rather than AMD per se. It is also interesting to note SDD alone also had a lower GRS than the other drusen related features though still significantly associated in keeping with recent genetic studies showing significant associations with the major AMD susceptibility loci.^37^

### Limitations

Our study suffers from several limitations. First, the response rate for those who attended the health assessment was moderate, but we implemented appropriate weighting strategies to mitigate the effects of such bias. Nonetheless, when weighted and unweighted prevalence estimates were compared (table 1), the differences were minor suggesting that the cohort demographic structure closely matched that of the general population. Second, we investigated a large number of potential risk factors a process that can increase the risk of false positive results. We therefore took a highly conservative approach in the creation of the multivariable models and interpreted the findings in terms of effect size and biological plausibility rather than explicit p value cut-offs. Third, we did not grade for intraretinal hyper-reflective foci an OCT feature, which is now considered a biomarker of deteriorating retinal pigment epithelium (RPE) health and a predictor for progression to late AMD.^38^

## Conclusions

This study provides further insight into the prevalence and risk factors of AMD and AMD features using multiple imaging modalities. Interestingly, the correlation between the Beckman classification and our findings from UWF imaging provide evidence that on a pragmatic level that the former continues to have validity. It highlights the benefits of using a multimodal approach in future epidemiological studies but also the challenges in interpretating findings that can be compared with previous colour only studies. New severity stage systems that incorporate AMD-based OCT features are urgently needed.

## Data Availability

Data are available on reasonable request. Details on data access policy available at https://www.qub.ac.uk/sites/NICOLA/InformationforResearchers/.
