# Supplementary material for: Prevalence and risk factors for age-related macular degeneration in a population-based cohort study of older adults in Northern Ireland using multimodal imaging: NICOLA Study
Source: Br J Ophthalmol. 2022 Oct 10;107(12):1873–9. doi: 10.1136/bjo-2021-320469 (PMC10715512; doi:10.1136/bjo-2021-320469)
Supplement: Supplementary data [file bjo-2021-320469supp001.pdf]

Prevalence and risk-factors for age-related macular degeneration in a population-based cohort study of older adults in Northern Ireland using multi-modal imaging- NICOLA Study

Ruth E. Hogg et al.

Supplementary Methods

Retinal image acquisition

Participants were given the option of having the pupils of neither (2592/6840, 37.9%) one eye (right eye =832/6840, 12.2%, left eye = 1424/6840, 20.8%) or both eyes dilated (1992/6840, 29.1%) using 1% tropicamide. Multi-modal retinal imaging was undertaken with three capture systems (i) standard colour fundus photography (CFP) using the Canon CX-1 Fundus Camera (Canon U.S.A., Inc.), (ii) wide-field color on the 200 TX, (Optos plc, Dunfermline, UK) (iii) blue light autofluorescence and (iii) Autofluorescence (AF), MultiColor (MC) and SD-OCT on the Spectralis (Heidelberg Engineering, Heidelberg, Germany). CFP of the disc and macula and AF were captured with a 50° field of view. A single non-stereo unsteered pseudocolour UWF image was obtained on the Optomap. All images except multicolour (MC) and AF were captured with or without pupillary dilation. MC and AF were only obtained if the participant agreed to pupillary dilation.

Macular scans were centred on the fovea, with an ART setting of 9. Images were 768x768 pixels with a resolution of approximately 11µm per pixel. Each OCT volume scan was composed of 61 horizontal B-scan lines with a spacing of approximately 125µm on a 30° x 25° (horizontal x vertical) scan angle and was acquired using active eye tracking and automatic real-time mean imaging. Enhanced Depth Imaging (EDI) mode was employed at an ART setting of 40 for

macula centered scans. MultiColor images were centred on the fovea, with an ART setting of 10 (30°).

All examination procedures including image acquisition were performed by trained personnel who applied standardised validated protocols. Image quality was monitored during the data collection period by an accredited ophthalmic technician who provided remedial training if necessary.

Physical measurements

Visual acuity was recorded as the number of letters correctly identified from either a 4m chart or a 1m chart while wearing their usual correction using a pinhole occluder. Autorefraction was measured using the auto-refractometer (Shin Nippon Accuref K-900, Rexxam, Japan).

Waist and hip measurements, as well as weight and height were obtained with participants wearing light clothing. Body mass index (BMI) was calculated as weight in kilograms divided by height in metres squared. Blood pressure was measured using an OMRON™ (Omron Healthcare Co, Ltd., Kyoto, Japan) digital oscillometric blood pressure monitor (Model M10-IT) with the mean calculated from three separate readings while seated at rest. Resting heart rate, in beats per minute, were also recorded using the digital blood pressure monitor. Cognitive Status was measured using the Montreal Cognitive Assessment (MOCA)[1] and the mini mental-state examination (MMSE)[2].

Self-report

Medical history was obtained during the CAPI and covered a range of common illnesses. Information on past physical activity was obtained using the short form of

the International Physical Activity Questionnaire (IPAQ) which has been validated for assessing activity levels in older adults.[3] Participants were asked to report the time they spent walking and in moderate and vigorous physical activity per week and the total was summed.

Blood-based Biomarkers

Blood samples for biochemical biomarkers were collected in tubes appropriate for specific tests including EDTA / clot activator. Whole blood (50.4 ml) samples were obtained from 3082 participants. The full list of plasma serum biomarkers that were analysed are shown in the supplementary methods and biochemical analyses for plasma, serum and DNA. Only those considered relevant for AMD were included in the risk factor analysis.

Genetic Analysis

DNA was extracted from buffy coats with DNA quantified using PicoGreen and normalised to 200 ng/uL aliquots with 0.1 and stored at -80°C. Samples were genotyped by Eurofins Scientific (Eurofins Genomics: https://www.eurofinsgenomics.eu). Genotype data (n = 551,839 markers directly typed) was generated using the Illumina Infinium CoreExome-24[4] for high-throughput screening on an iScan. GenomeStudio® Genotyping Module was used as the calling algorithm, using the Genome Reference Consortium Human Build 37 (GRCh37). QC of the genotyped data was performed in PLINK 1.90 beta[5]. Variants with a call rate ≥ 98 %, Hardy-Weinberg equilibrium $p>10^{-6}$ and Minor Allele Frequency (MAF) $<$ 0.0001 were removed.

Files were prepared for imputation using the "*HRC/1KG Imputation Preparation and Checking Tool*"[6], and then imputed to the 1000 Genomes Phase3 v5 (1KGP3) and Haplotype Reference Consortium (HRC) r1.1 2016 reference panels using the Michigan Imputation Server[7] (53). Of the 52 SNPs reported by Fritsche et al (2016), [8] 7 did not meet the quality criteria (rs121913059, rs142450006, rs116503776, rs144629244, rs114254831, rs79037040, rs71507014). Genetic risk scores (GRS) were calculated by multiplying the conditional β value of the AMD risk variant with the allele dosage and then summing all calculations as per Colijn et al (2020).[9] The GRS provides a continuous variable which represents an overall genetic risk for AMD and which was derived from the genome-wide association study based on genetic data from 16,144 patients with AMD and 17,832 controls and identified 52 common and rare variants distributed across 34 loci.[8]

Retinal Image grading

Trained graders within the Network of Ophthalmic Reading Centres UK (NetWORC UK) undertook color, MC and OCT grading to a prespecified protocol. UWF images were graded by a single research fellow with review and arbitration by retinal experts (UC and TP). Color images were displayed on a bespoke grading platform (Oculab V3.7.98.0) with screen settings at the highest available resolution at 1920 x 1080. MC and OCT images were graded using the Heidelberg Eye Explorer Viewing Module 6.0.13.0 (Heidelberg Engineering, Heidelberg, Germany). Following image acquisition, Optomap images underwent transformation using Optos stereographic projection software (ProView), which takes into account the optical imaging system and the ocular geometry to map each pixel to a consistent spherical geometry[10]. Inaccurately projected images due to quality and/or gaze angle were re-projected manually using ImageJ software version 1.50 (National Institutes of Health, Bethesda,

MD; available at http://rsb.info.nih.gov/ij/index.html), finding the x- and y- coordinates of the fovea.

Graders were certified for study specific procedures using a standard set of images with performance checked every 3 months for drift. The average certification score for graders was 87.9% concordance. Within the grading protocol there were separate questions for assessing AMD phenotypes on *en face* images (Color, MC or AF) and OCT B scans.

Graders used color images to identify drusen within and outside the ETDRS grid. When present within the grid the most frequent size of drusen present within the grid (<63µm, 63-125µm >125µm or crystalline) was recorded. The presence of exudative AMD was based on the presence of any of the following features: choroidal neovascularisation, serous retinal fluid (SRF), intra-retinal or sub-retinal exudates, haemorrhage, sub-retinal fibrosis or atrophic scar, retinal pigment epithelial detachment (PED). Geographic Atrophy (GA) was defined as a sharply defined drop-out of the RPE and choriocapillaris (which must be at least 175 µm in diameter), exposing the underlying large choroidal vessels and with well-defined margins and scalloped edges. The presence of reticular pseudodrusen (RPD) was assessed using color, infra-red, autofluorescence and multicolor images if available. RPD only had to be visible on one *en face* modality to be considered present.

Ultra-widefield image grading

The Manchester grid (MG) tool and Optos V2 Vantage Dx Review 2.5.0.135 were used in combination to grade the UWF images. The Manchester grid was initially developed for grading retinal ischaemia in fluorescein angiograms by Professor Paulo Stanga at eh University of Manchester. It is composed of a grid of x and y -

coordinates centred on the fovea (0,0). The MG covers the retina with 754 squares with each square being roughly one optic disc in size (1.77mm^2) (Figure 1 below). It enables the spatial of location of the lesion and the approximate size to be recorded. UWF images were loaded onto the MG tool, each square-like box approximately one optic disc in size (1.77mm^2). x- and y- coordinates were centred on the fovea (co-ordinates 0,0) to enable approximate size and location of pathology to be derived. The ungradable area was also recorded (defined as occurring when 80% of the square was not visible). AMD features including hard or soft drusen, hyperpigmentation and geographic atrophy were graded using standard definitions based on the Wisconsin Age-Related Maculopathy Grading Scheme (WARMGS). UWFI were graded without access to clinical information by a single research fellow involved in the study (NQ). In the event of uncertainty adjudication was provided by two expert clinicians (TP & UC). Intra-grader reliability was assessed in 120 consecutive images (60 participants) by NQ, blinded to the initial outputs and a time difference of 6 months between first and second grading. For lesions graded, Kappa values ranged from 0.85-1.00 indicating almost perfect agreement.

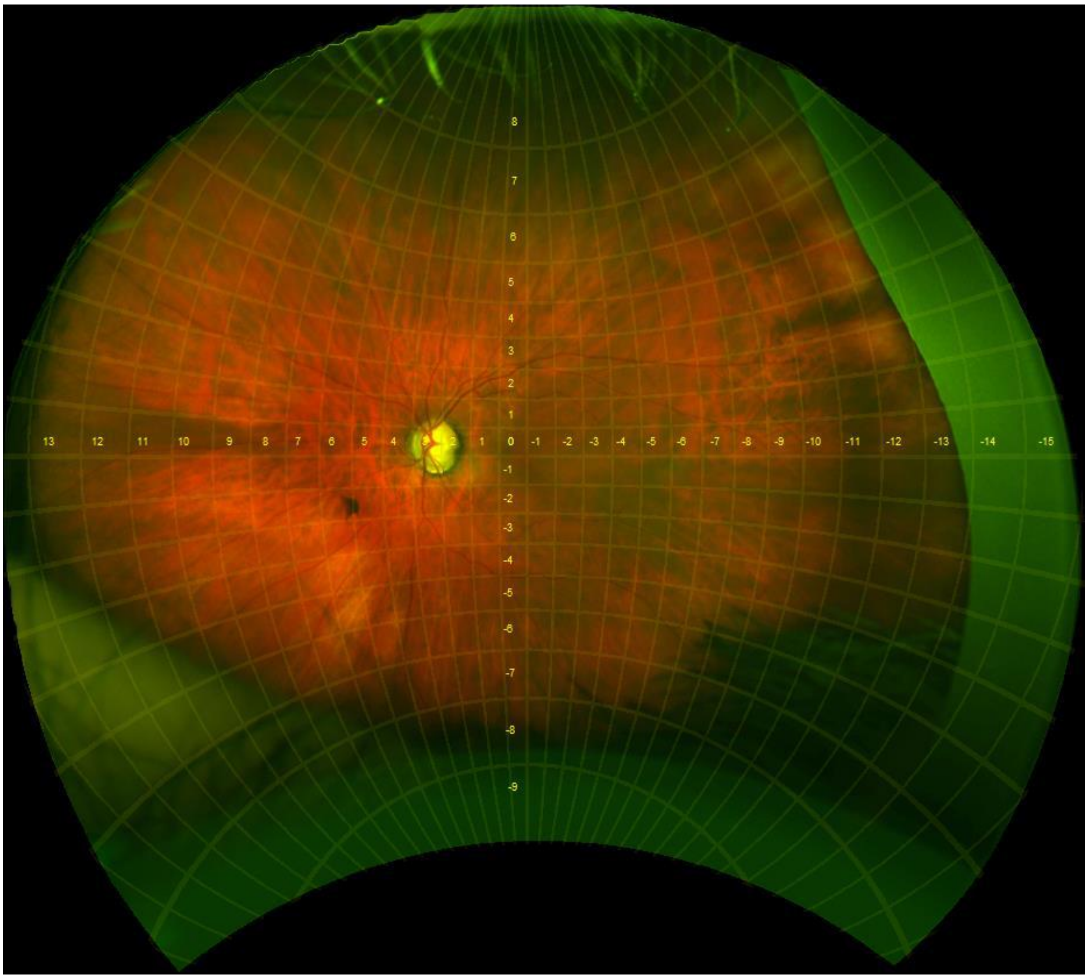

Figure 1 The Manchester Grid. The retina is covered in 754 optic disc sized boxes with x- and y- co-ordinates centered on the fovea.

To explore the spatial distribution of different retinal lesions the MG was divided into the centre region, mid-periphery region and far-periphery region. The centre region was defined by analysing corresponding colour fundus photography images and recording the x- and y- co-ordinates where the area on UWFI matched the colour fundus photography images on a subset of 20 images. The mid-periphery was defined as the x- and y- co-ordinates corresponding to the location of the vortex

veins on a subset of 20 UWFI. The far-periphery was defined as the area beyond the mid-periphery.

These corresponded to the following cut points on the MG:

Left eye

Centre: X : >= -4 and < 5 **Y** : >= -3 and <= 3

Mid-periphery: **X** : >= -9 and <= 10 **Y** : >= -5 and <= 5

Far-periphery: X : < -9 and >10 **Y** : < -5 and > 5

Right eye

Centre: X : >= -5 and < 4 **Y** : >= -3 and <= 3

Mid-periphery: **X** : >= -10 and <= 9 **Y** : >= -5 and <= 5

Far-periphery: X : < -10 and >9 **Y** : < -5 and > 5

OCT image grading

All the component B scans of the volume scan were scrutinized and in the absence of any abnormalities recorded as normal. Drusen were defined as the presence of RPE deformation or thickening resulting in irregularities and undulations of the layer. SDD was defined as subretinal accumulation of drusenoid material and categorised into ‘discrete’, ‘diffuse’ or ‘both’ according to Suzuki et al, (2014)[11]. Complete retinal pigment epithelium and outer retinal atrophy (cRORA) was deemed to be present if there was: *(i)*Increased signal transmission into the choroid *(ii)*RPE band thinned or missing *or (iii)* Outer nuclear layer thinned or missing, (iv) inner nuclear layer and or outer plexiform layer subsidence[12]. Graders assessed the thickness of the choroid from EDI B scans passing through the foveal avascular zone and assigned a category to the eye as thin, normal or thick compared to a set of exemplar images.

Discrepancies in grading were checked by the senior grader and cases of late-stage AMD and other pathology by the reading center clinicians. (UC and TP).

BMJ Publishing Group Limited (BMJ) disclaims all liability and responsibility arising from any reliance placed on this supplemental material which has been supplied by the author(s)

Covariate selection and categorisation

Known risk factors were selected based on published literature. Age was divided into five categories: <55years, 55-64 years, 65-74 years, 75-84 years and > 85 years. Hypertension categories were normal (systolic blood pressure (SBP) <120 mm Hg and diastolic blood pressure (DBP) <80 mm Hg); elevated (SBP 120-129 mm Hg and DBP <80 mm Hg); stage 1 hypertension (SBP 130-139 mm Hg or DBP 80-89 mm Hg); stage 2 hypertension (SBP ≥140 mm Hg or DBP ≥90 mm Hg). Smoking status was categorised as never, ex-smokers and current. Physical Activity: classified as meeting (≥150 minutes per week) or not meeting (<150 minutes per week) current physical activity guidelines for older adults.[13] Spherical equivalent was calculated using the standard formula (Spherical equivalent= sphere + (cylinder/2)). Lens status was categorised as pseudophakic, phakic cataract present and clear lens. In addition to self-report of diabetes a diabetes case status variable was based on self-report of Diabetes in the CAPI or health assessment, a record of taking diabetes related medication or an HbA1c measurement ≥48mmol/mol.[23]

Main outcome categorisation

The main outcome was the AMD stage based on the Beckman clinical classification as stage 0 (No drusen or pigmentary abnormalities), stage 1 (small drusen ≤63µm), stage 2 (drusen >63µm and ≤125µm and no pigmentary abnormalities), stage 3 (medium drusen with pigmentary abnormalities or large drusen >125µm with or without pigmentary abnormalities) and stage 4 (neovascular AMD and/or geographic atrophy).[14] The arbitrated AMD stage was used to assign each eye to one of the 4 Beckman stages. The person level classification based on the stage of the worse eye was used in determining prevalence and for risk factor association analysis (stages 0 and 1: no AMD vs. stages 2, 3 and 4: AMD).

Hogg RE, *et al. Br J Ophthalmol* 2023; 107:1873–1879. doi: 10.1136/bjo-2021-320469

Multiple imputation

Multiple regression was complicated by the high proportion of missing values for some variables (Table S1). At the individual level, across the full set of predictor variables used for these analyses, 1856 were complete. Using complete cases would have substantially increased the risk of bias and reduced the power of our analysis. Therefore, multiple imputation was conducted to enable the maximum number of records to be used. This was done using the *mice* package in *R*[15 16].

Multiple imputation was performed at the person level to allow for correlations between eyes within individuals when imputing missing eye-level values. Each individual formed a row in the dataset and missing values for one eye were imputed accounting for the values observed in the other eye.

The MICE algorithm proceeds by constructing imputation models for variables with missing values that include values imputed for other variables in earlier iterations. Predictive mean matching, logistic regression and multinomial logit models were used for imputing continuous, binary and multinomial variables, respectively.

As each person record consisted of the person level risk factors and those from each eye, there was a large number of potential predictor variables for each imputation model. To speed up algorithm convergence, predictor variables were screened prior to imputation, with those showing an absolute correlation with the target variable < 0.1 removed from the imputation model as they contributed little usable information.

The default setting of mice is to use all other variables to predict a given target variable. This is not sensible if there are structural associations between the variables. For example, hypertension categories were derived from continuous measures of diastolic blood pressure and systolic blood pressure, leading to collinearity. Therefore, derived variables and those that

were a linear combination of others (e.g. total cholesterol as a combination of LDL, HDL and triglycerides) were excluded to produce the final set of variables for imputation and modelling.

5 imputations were performed, iterating until the chains were well mixed indicating convergence. Multiple regression models were fitted to the multiple imputed datasets produced at iteration number 70 and the resulting estimates pooled according to Rubin's rules.[17] Multiple regression results reported for the individual level dataset were all produced in this manner.

Table S1. Distribution of missingness by variable for those variables with at least one missing record.

Variable	Missing records	% missing
Genetic Risk Score	615	18.2
Lpa mg/dL	605	17.9
Vitamin D ng/mL	398	11.8
Spherical Equivalent (Diopters) – Right eye	339	10.0
Spherical Equivalent (Diopters) – Left eye	331	9.8
hsCRP mg/L	193	5.7
LDL mmol/L	185	5.5
HDL mmol/L	185	5.5
Triglyceride mmol/L	185	5.5
Creatinine mg/dL	185	5.5
ApoA1 g/L	185	5.5
ApoB g/L	185	5.5
Choroidal thickness – Left eye	124	3.7
Evidence of epiretinal membrane – Left eye	114	3.4
Evidence of vitreomacular adhesion – Left eye	114	3.4
Choroidal thickness – Right eye	64	1.9
Evidence of epiretinal membrane – Right eye	57	1.7
Evidence of vitreomacular adhesion – Right eye	57	1.7
Physical activity	30	0.9

Variable	Missing records	% missing
MOCA Score < 26	26	0.8
Cataract	19	0.6
Hypertension Category	14	0.4
Body Mass Index	8	0.2
Education	3	0.1
Smoker	3	0.1
Waist Hip Ratio	1	0.0

1. Nasreddine ZS, Phillips NA, Bedirian V, et al. The Montreal Cognitive Assessment, MoCA: a brief screening tool for mild cognitive impairment. *J Am Geriatr Soc* 2005;53(4):695-9. doi: 10.1111/j.1532-5415.2005.53221.x [published Online First: 2005/04/09]
2. Folstein MF, Robins LN, Helzer JE. The Mini-Mental State Examination. *Arch Gen Psychiatry* 1983;40(7):812. doi: 10.1001/archpsyc.1983.01790060110016 [published Online First: 1983/07/01]
3. Grimm EK, Swartz AM, Hart T, et al. Comparison of the IPAQ-Short Form and accelerometry predictions of physical activity in older adults. *J Aging Phys Act* 2012;20(1):64-79. doi: 10.1123/japa.20.1.64 [published Online First: 2011/12/23]
4. Illumina I. InfiniumTM CoreExome-24 v1.3 BeadChip 2018 [Available from: https://emea.illumina.com/content/dam/illumina-marketing/documents/products/datasheets/datasheet_human_core_exome_beadchip.pdf accessed 11/12/2020 2020.
5. Chang CC, Chow CC, Tellier LC, et al. Second-generation PLINK: rising to the challenge of larger and richer datasets. *Gigascience* 2015;4:7. doi: 10.1186/s13742-015-0047-8 [published Online First: 2015/02/28]
6. Raynor W. HRC or 1000G Imputation preparation and checking 2020 [Available from: https://www.well.ox.ac.uk/~wrayner/tools/ accessed 12/12/2020 2020.
7. Das S, Forer L, Schonherr S, et al. Next-generation genotype imputation service and methods. *Nat Genet* 2016;48(10):1284-87. doi: 10.1038/ng.3656 [published Online First: 2016/08/30]
8. Fritsche LG, Igl W, Bailey JN, et al. A large genome-wide association study of age-related macular degeneration highlights contributions of rare and common variants. *Nat Genet* 2016;48(2):134-43. doi: 10.1038/ng.3448 [published Online First: 2015/12/23]

9. Colijn JM, Meester-Smoor M, Verzijden T, et al. Genetic Risk, Lifestyle, and Age-Related Macular Degeneration in Europe: The EYE-RISK Consortium. *Ophthalmology* 2020 doi: 10.1016/j.ophtha.2020.11.024 [published Online First: 2020/12/01]
10. Croft DE, van Hemert J, Wykoff CC, et al. Precise montaging and metric quantification of retinal surface area from ultra-widefield fundus photography and fluorescein angiography. *Ophthalmic Surg Lasers Imaging Retina* 2014;45(4):312-7. doi: 10.3928/23258160-20140709-07 [published Online First: 2014/07/19]
11. Suzuki M, Sato T, Spaide RF. Pseudodrusen subtypes as delineated by multimodal imaging of the fundus. *Am J Ophthalmol* 2014;157(5):1005-12. doi: 10.1016/j.ajo.2014.01.025 [published Online First: 2014/02/08]
12. Sadda SR, Guymer R, Holz FG, et al. Consensus Definition for Atrophy Associated with Age-Related Macular Degeneration on OCT: Classification of Atrophy Report 3. *Ophthalmology* 2018;125(4):537-48. doi: 10.1016/j.ophtha.2017.09.028 [published Online First: 2017/11/07]
13. Bull FC, Al-Ansari SS, Biddle S, et al. World Health Organization 2020 guidelines on physical activity and sedentary behaviour. *Br J Sports Med* 2020;54(24):1451-62. doi: 10.1136/bjsports-2020-102955 [published Online First: 2020/11/27]
14. Ferris FL, 3rd, Wilkinson CP, Bird A, et al. Clinical classification of age-related macular degeneration. *Ophthalmology* 2013;120(4):844-51. doi: 10.1016/j.ophtha.2012.10.036 [published Online First: 2013/01/22]
15. Team RC. R: a language and environment for statistical computing. Vienna, Austria.: R Foundation for Statistical Computing 2019.
16. van Buuren S, Groothuis-Oudshoorn, K. . mice: Multivariate Imputation by Chained Equations in R. *Journal of Statistical Software* 2011;45:1-67.
17. Heymans M. EI. Applied Missing Data Analysis with SPSS and (R) Studio, 2019.
