# Supplementary material for: Prevalence and risk factors for age-related macular degeneration in a population-based cohort study of older adults in Northern Ireland using multimodal imaging: NICOLA Study
Source: Br J Ophthalmol. 2022 Oct 10;107(12):1873–9. doi: 10.1136/bjo-2021-320469 (PMC10715512; doi:10.1136/bjo-2021-320469)
Supplement: Supplementary data [file bjo-2021-320469supp002.pdf]

Supplementary material

Supplementary Table 1 Participant characteristics by presence of colour, OCT and ultra-widefield grading.

Participant characteristics	Colour fundus photography			Optical Coherence Tomography			Ultra-wide field imaging		
	At least 1 gradable eye n=3265	No images or both ungradable n=121	P*	At least 1 gradable eye n=3344	No images or both ungradable n=42	P*	At least 1 gradable eye n=3214	No images or both ungradable n=172	P*
**Age (SD)**	63.5 (8.9)	69.3 (9.6)	<0.001	63.6 (9)	68.6 (11.4)	0.007	63.4 (8.9)	68.6 (9.8)	<0.001
**Female (%)**	1706 (52.3)	59 (48.8)	0.508	1744 (52.2)	21 (50.0)	0.903	1669 (51.9)	96 (55.8)	0.360
**Genetic Risk Score (SD)**	10.9 (1.2)	11.1 (1.2)	0.120	10.9 (1.2)	10.9 (1.3)	0.822	10.9 (1.2)	11.1 (1.1)	0.25
**Waist Hip Ratio (SD)**	0.9 (0.1)	1 (0.8)	0.234	0.9 (0.1)	1.1 (1.3)	0.328	0.9 (0.1)	1 (0.6)	0.29
**Body Mass Index (SD)**	28.9 (5)	29.2 (5.8)	0.485	28.9 (5.1)	28.6 (4.8)	0.743	28.9 (5)	28.8 (5.6)	0.78
**Systolic Blood Pressure mmHg (SD)**	133.8 (32.3)	138.9 (43.6)	0.202	133.8 (31.3)	152 (94.4)	0.217	133.6 (30.7)	141.1 (59.5)	0.11
**Diastolic Blood Pressure mmHg (SD)**	83.3 (31.1)	84.5 (43.8)	0.781	83.2 (29.8)	100.8 (99.3)	0.257	83.1 (29.2)	88 (61.7)	0.30
**Pulse 10's beats per minute (SD)**	7.8 (7.3)	7.7 (4.4)	0.888	7.7 (7.2)	9.6 (10)	0.227	7.7 (7.1)	8.4 (9.4)	0.33

**Spherical Equivalent (Diopters) (SD)**	0.7 (2.4)	0.5 (2.7)	0.469	0.7 (2.4)	1 (1.6)	0.434	0.8 (2.3)	0.2 (3.1)	0.03
**Self-report**									
**Cardiovascular Disease (%)**	455 (13.9)	23 (19.0)	0.150	467 (14.0)	11 (26.2)	0.042	444 (13.8)	34 (19.8)	0.038
**Chronic lung disease (%)**	109 (3.3)	5 (4.1)	0.827	112 (3.3)	2 (4.8)	0.941	104 (3.2)	10 (5.8)	0.108
**Asthma (%)**	309 (9.5)	14 (11.6)	0.537	320 (9.6)	3 (7.1)	0.789	301 (9.4)	22 (12.8)	0.175
**Cognitive problems (%)**	19 (0.6)	3 (2.5)	0.048	20 (0.6)	2 (4.8)	0.018	18 (0.6)	4 (2.3)	0.020
**Arthritis (%)**	912 (27.9)	46 (38.0)	0.021	946 (28.3)	12 (28.6)	1.000	904 (28.1)	54 (31.4)	0.401
**Cancer (%)**	260 (8.0)	11 (9.1)	0.781	263 (7.9)	8 (19.0)	0.018	254 (7.9)	17 (9.9)	0.430
**Parkinson's (%)**	10 (0.3)	0 (0.0)	1.000	10 (0.3)	0 (0.0)	1.000	9 (0.3)	1 (0.6)	1.000
**Diabetes (%)**	290 (8.9)	16 (13.2)	0.140	301 (9.0)	5 (11.9)	0.703	282 (8.8)	24 (14.0)	0.030
**Education**									
**Primary or less (%)**	463 (14.2)	27 (22.3)	0.081	480 (14.4)	10 (23.8)	0.357	456 (14.2)	34 (19.8)	0.134
**Secondary (%)**	2027 (62.1)	71 (58.7)		2076 (62.1)	22 (52.4)		2004 (62.4)	94 (54.7)	
**Degree or higher (%)**	772 (23.6)	23 (19.0)		785 (23.5)	10 (23.8)		751 (23.4)	44 (25.6)	
**Missing (%)**	3 (0.1)	0 (0.0)		3 (0.1)	0 (0.0)		3 (0.1)	0 (0.0)	
**Smoker**									
**Never (%)**	1725 (52.8)	63 (52.1)	0.980	1769 (52.9)	19 (45.2)	0.545	1696 (52.8)	92 (53.5)	0.706
**Former (%)**	1210 (37.1)	45 (37.2)		1235 (36.9)	20 (47.6)		1196 (37.2)	59 (34.3)	

**Current (%)**	327 (10.0)	13 (10.7)		337 (10.1)	3 (7.1)		319 (9.9)	21 (12.2)	
**Missing (%)**	3 (0.1)	0 (0.0)		3 (0.1)	0 (0.0)		3 (0.1)	0 (0.0)	
**Physical Activity**									
**≥150 minutes per week (%)**	2164 (66.3)	68 (56.2)	0.028	2209 (66.1)	23 (54.8)	0.219	2130 (66.3)	102 (59.3)	0.169
**< 150 minutes per week (%)**	1071 (32.8)	53 (43.8)		1105 (33.0)	19 (45.2)		1056 (32.9)	68 (39.5)	
**Cataract**									
**Never (%)**	2645 (81.0)	75 (62.0)	<0.001	2696 (80.6)	24 (57.1)	<0.001	2622 (81.6)	98 (57.0)	<0.001
**Present (%)**	403 (12.3)	26 (21.5)		421 (12.6)	8 (19.0)		382 (11.9)	47 (27.3)	
**Surgery (%)**	200 (6.1)	18 (14.9)		212 (6.3)	6 (14.3)		194 (6.0)	24 (14.0)	
**Missing (%)**	17 (0.5)	2 (1.7)		15 (0.4)	4 (9.5)		16 (0.5)	3 (1.7)	
**Hypertension category**									
**SBP<120 & DBP<80 (%)**	742 (22.7)	22 (18.2)	0.013	755 (22.6)	9 (21.4)	0.803	725 (22.6)	39 (22.7)	0.880
**120≤SBP<130 & DBP<80 (%)**	366 (11.2)	10 (8.3)		370 (11.1)	6 (14.3)		357 (11.1)	19 (11.0)	
**130≤SBP<140 Or 80≤DBP< 90 (%)**	1399 (42.8)	45 (37.2)		1424 (42.6)	20 (47.6)		1376 (42.8)	68 (39.5)	
**SBP≥140 Or DBP≥90 (%)**	744 (22.8)	44 (36.4)		781 (23.4)	7 (16.7)		743 (23.1)	45 (26.2)	
**Missing (%)**	14 (0.4)	0 (0.0)		14 (0.4)	0 (0.0)		13 (0.4)	1 (0.6)	

**Diabetes**									
**No (%)**	2844 (87.1)	102 (84.3)	0.445	2912 (87.1)	34 (81.0)	0.346	2808 (87.4)	138 (80.2)	0.0095
**Yes (%)**	421 (12.9)	19 (15.7)		432 (12.9)	8 (19.0)		406 (12.6)	34 (19.8)	
**Alcohol intake**									
**0 units/day (%)**	987 (30.2)	43 (35.5)	0.359	1012 (30.3)	18 (42.9)	0.209	965 (30.0)	65 (37.8)	0.097
**0 < units/day < 4 (%)**	2018 (61.8)	67 (55.4)		2064 (61.7)	21 (50.0)		1990 (61.9)	95 (55.2)	
**≥ 4 units/day (%)**	260 (8.0)	11 (9.1)		268 (8.0)	3 (7.1)		259 (8.1)	12 (7.0)	
**Cognitive**									
**MMSE Score < 24 (%)**	64 (2.0)	2 (1.7)	1.000	65 (1.9)	1 (2.4)	1.000	61 (1.9)	5 (2.9)	0.516
**MOCA Score < 26 (%)**	1340 (41.0)	58 (47.9)	0.0011	1377 (41.2)	21 (50.0)	0.223	1310 (40.8)	88 (51.2)	0.0061
**MOCA Score missing (%)**	22 (0.7)	4 (3.3)		25 (0.7)	1 (2.4)		23 (0.7)	3 (1.7)	
**Serum**									
**hsCRP mg/L (SD)**	3.2 (5.3)	4.6 (7.7)	0.057	3.3 (5.4)	4.2 (4.6)	0.227	3.3 (5.4)	3.3 (4.7)	1.00
**LDL mmol/L (SD)**	3.4 (1.1)	3 (1)	<0.001	3.4 (1.1)	3.3 (1)	0.574	3.4 (1.1)	3.2 (1.1)	0.01
**HDL mmol/L (SD)**	1.6 (0.4)	1.6 (0.5)	0.720	1.6 (0.4)	1.6 (0.4)	0.365	1.6 (0.4)	1.6 (0.4)	0.87
**Vitamin D ng/mL (SD)**	22.2 (10.6)	23.3 (10.6)	0.308	22.2 (10.7)	21.4 (9.1)	0.578	22.2 (10.6)	23.2 (10.9)	0.24
**Lpa mg/dL (SD)**	20.6 (22.4)	16.9 (18)	0.043	20.5 (22.3)	23.6 (23.7)	0.457	20.6 (22.4)	19 (19.9)	0.37

**Triglyceride mmol/L (SD)**	3.7 (2.1)	3.6 (1.7)	0.371	3.7 (2.1)	3.8 (2.4)	0.837	3.7 (2.1)	3.5 (1.6)	0.08
**Creatinine mg/dL (SD)**	0.9 (0.3)	1 (0.6)	0.066	0.9 (0.3)	0.9 (0.3)	0.262	0.9 (0.3)	1 (0.5)	0.07
**ApoA1 g/L (SD)**	1.7 (0.3)	1.7 (0.3)	0.993	1.7 (0.3)	1.7 (0.3)	0.439	1.7 (0.3)	1.7 (0.3)	0.72
**ApoB g/L (SD)**	1 (0.3)	1 (0.2)	0.002	1 (0.3)	1 (0.3)	0.776	1 (0.3)	1 (0.2)	0.07

* Statistical comparisons performed using independent sample t-tests (continuous variables) or Chi-squared tests (categorical variables).

Supplementary Table 2 Prevalence of AMD % (95% CI) using the Beckman Clinical Classification by age category (Person level analysis). Unweighted and weighted estimates are included.

Unweighted

Age	Number at risk	0	1	2	3	4
**Males**						
<55	228	60.1 (53.4, 66.5)	24.1 (18.7, 30.2)	11.0 (7.2, 15.8)	4.8 (2.4, 8.5)	0.0
55-64	564	63.3 (59.2, 67.3)	19.7 (16.5, 23.2)	11.2 (8.7, 14.1)	5.9 (4.1, 8.1)	0.0
65-74	556	62.6 (58.4, 66.6)	15.5 (12.6, 18.7)	11.7 (9.1, 14.7)	9.0 (6.7, 11.7)	1.3 (0.5, 2.6)
75-84	182	51.1 (43.6, 58.6)	16.5 (11.4, 22.7)	15.4 (10.5, 21.5)	14.3 (9.5, 20.2)	2.7 (0.9, 6.3)
≥85	29	51.7 (32.5, 70.6)	10.3 (2.2, 27.4)	10.3 (2.2, 27.4)	17.2 (5.8, 35.8)	10.3 (2.2, 27.4)
**Females**						
<55	347	65.1 (59.9, 70.1)	23.3 (19.0, 28.2)	8.4 (5.7, 11.8)	3.2 (1.6, 5.6)	0.0
55-64	693	65.7 (62.0, 69.2)	17.5 (14.7, 20.5)	13.0 (10.6, 15.7)	3.8 (2.5, 5.4)	0.1 (0.0, 0.8)
65-74	505	55.6 (51.2, 60.0)	16.6 (13.5, 20.2)	18.4 (15.1, 22.1)	8.5 (6.2, 11.3)	0.8 (0.2, 2.0)
75-84	143	59.4 (50.9, 67.6)	7.0 (3.4, 12.5)	18.9 (12.8, 26.3)	11.9 (7.1, 18.4)	2.8 (0.8, 7.0)

Unweighted

Age	Number at risk	0	1	2	3	4
≥85	18	55.6 (30.8, 78.5)	11.1 (1.4, 34.7)	5.6 (0.1, 27.3)	16.7 (3.6, 41.4)	11.1 (1.4, 34.7)

Supplementary Table 3 Prevalence of AMD features by age category and sex (Eye level analysis, weighted).

	En face grading							OCT grading						
Age	Number at risk	Pigmentary Irregularities	Drusen (any size)	Drusen ≥63µm	Drusen ≥125µm	Reticular Drusen	GA	Number at risk	Classical Drusen	SDD	Focal Atrophy	CNV	Vitelliform	Any AMD
**Males**														
<55	447	1.1 (0.4, 2.6)	28.4 (23.5, 33.7)	9.2 (6.3, 12.8)	1.8 (0.7, 3.8)	0.9 (0.2, 2.7)	0.0	462	19.7 (15.5, 24.4)	15.6 (12.0, 19.8)	5.0 (2.8, 8.0)	0.0	0.0	9.8 (6.9, 13.5)
55-64	1087	1.7 (0.9, 2.7)	24.9 (22.0, 28.0)	9.9 (8.0, 12.2)	2.0 (1.2, 3.2)	1.4 (0.7, 2.4)	0.0	1144	20.9 (18.2, 23.8)	19.8 (17.2, 22.6)	2.3 (1.4, 3.4)	0.0	0.1 (0.0, 0.5)	11.0 (9.0, 13.4)
65-74	1046	3.1 (1.9, 4.6)	27.9 (24.7, 31.3)	13.8 (11.4, 16.4)	4.2 (2.9, 5.9)	2.5 (1.5, 3.9)	0.9 (0.3, 1.9)	1126	27.2 (24.0, 30.5)	25.6 (22.6, 28.7)	5.2 (3.7, 6.9)	0.1 (0.0, 0.5)	0.6 (0.2, 1.5)	15.5 (13.0, 18.3)
75-84	329	1.8 (0.7, 3.9)	40.7 (34.2, 47.5)	24.6 (19.0, 31.0)	9.7 (6.1, 14.5)	7.3 (4.1, 11.8)	0.9 (0.2, 2.6)	371	46.1 (39.5, 52.8)	44.7 (38.4, 51.2)	7.0 (4.2, 10.8)	1.2 (0.3, 3.1)	0.5 (0.0, 3.0)	27.1 (21.2, 33.6)
≥85	52	7.7 (1.4, 22.0)	38.5 (22.7, 56.2)	23.1 (10.0, 41.5)	11.5 (3.7, 25.5)	15.4 (4.2, 35.2)	0.0	60	55.0 (37.2, 71.9)	50.0 (32.9, 67.1)	21.7 (10.0, 38.0)	5.8 (1.2, 16.0)	3.3 (0.1, 17.7)	28.8 (14.9, 46.4)

	En face grading							OCT grading						
Age	Number at risk	Pigmentary Irregularities	Drusen (any size)	Drusen ≥63µm	Drusen ≥125µm	Reticular Drusen	GA	Number at risk	Classical Drusen	SDD	Focal Atrophy	CNV	Vitelliform	Any AMD
**Females**														
<55	684	0.9 (0.3, 1.9)	24.4 (20.7, 28.5)	7.0 (4.9, 9.6)	1.0 (0.3, 2.5)	1.6 (0.6, 3.4)	0.0	689	14.1 (11.2, 17.4)	11.3 (8.8, 14.3)	0.9 (0.3, 2.1)	0.0	0.0	7.3 (5.2, 9.9)
55-64	1360	0.5 (0.2, 1.2)	24.5 (21.8, 27.4)	10.4 (8.6, 12.4)	1.8 (1.1, 2.8)	1.0 (0.5, 1.7)	0.0	1390	19.9 (17.5, 22.6)	17.1 (14.8, 19.5)	3.4 (2.3, 4.7)	0.1 (0.0, 0.4)	0.0	10.7 (8.9, 12.8)
65-74	957	1.7 (0.9, 2.8)	33.5 (30.0, 37.2)	19.3 (16.3, 22.6)	5.2 (3.6, 7.3)	5.3 (3.6, 7.5)	0.1 (0.0, 0.6)	1018	32.0 (28.5, 35.7)	29.3 (26.0, 32.8)	4.1 (2.8, 5.8)	0.4 (0.1, 1.3)	0.5 (0.1, 1.5)	20.6 (17.5, 23.9)
75-84	266	4.9 (2.3, 9.1)	36.5 (28.9, 44.6)	24.8 (18.5, 32.0)	6.4 (3.3, 10.9)	10.2 (6.0, 15.7)	1.5 (0.3, 4.6)	304	42.1 (35.1, 49.3)	43.4 (36.3, 50.7)	6.6 (3.6, 10.9)	0.4 (0.0, 2.1)	0.7 (0.0, 3.6)	27.4 (20.9, 34.8)
≥85	31	12.9 (2.4, 34.9)	41.9 (19.7, 66.9)	22.6 (6.1, 49.4)	6.5 (0.1, 32.1)	6.5 (0.1, 32.1)	0.0	37	54.1 (31.2, 75.7)	59.5 (36.1, 80.0)	8.1 (0.8, 28.4)	6.5 (0.7, 22.0)	0.0	29.0 (10.8, 54.2)

Supplementary Table 4 Risk factor associations with AMD (Beckman 2,3,4) versus Controls (Beckman 0,1).

Participant characteristics			Univariate		Age-sex adjusted models		Multivariable models		AMD Risk Score excluded	
	No AMD n=2590	AMD n=675	OR	P	OR	P	OR	P	OR	P
**Age (SD)**	62.7 (8.7)	66.2 (9.2)	1.04 (1.03, 1.05)	<0.001	1.07 (0.95, 1.19)	0.261	1.06 (0.95, 1.19)	0.297	1.06 (0.94, 1.18)	0.346
**Age^2**					1.00 (1.00, 1.00)	0.712	1.00 (1.00, 1.00)	0.742	1.00 (1.00, 1.00)	0.827
**Female (%)**	1355 (52.3)	351 (52.0)	0.99 (0.83, 1.17)	0.883	1.08 (0.91, 1.28)	0.383	1.01 (0.83, 1.22)	0.959	1.00 (0.82, 1.22)	0.977
**Genetic Risk Score (SD)**	0.4 (1.1)	0.7 (1.2)	1.20 (1.11, 1.30)	<0.001	1.20 (1.11, 1.30)	<0.001	1.21 (1.11, 1.31)	<0.001		
**Waist Hip Ratio (SD)**	0.9 (0.1)	0.9 (0.1)	0.87 (0.34, 2.19)	0.763	0.45 (0.13, 1.55)	0.206				
**Body Mass Index (SD)**	28.9 (5)	28.8 (5.3)	1.00 (0.98, 1.01)	0.616	1.00 (0.98, 1.02)	0.992				
**Resting heart rate (beats per minute) (SD)**	7.7 (7.2)	7.8 (7.6)	1.00 (0.99, 1.01)	0.716	1.00 (0.99, 1.01)	0.858				
**Self-report**										
**Cardiovascular Disease (%)**	346 (13.4)	109 (16.1)	1.25 (0.99, 1.58)	0.063	1.01 (0.79, 1.29)	0.946				

**Chronic lung disease (%)**	75 (2.9)	34 (5.0)	1.78 (1.17, 2.70)	0.006	1.60 (1.04, 2.44)	0.030	1.63 (1.06, 2.51)	0.026	1.52 (0.99, 2.34)	0.054
**Asthma (%)**	240 (9.3)	69 (10.2)	1.11 (0.84, 1.48)	0.450	1.14 (0.85, 1.52)	0.379				
**Cognitive problems (%)**	14 (0.5)	5 (0.7)	1.37 (0.49, 3.85)	0.544	1.18 (0.42, 3.36)	0.752				
**Arthritis (%)**	701 (27.1)	211 (31.3)	1.23 (1.02, 1.47)	0.031	1.05 (0.86, 1.27)	0.639				
**Cancer (%)**	209 (8.1)	51 (7.6)	0.93 (0.68, 1.28)	0.661	0.79 (0.57, 1.10)	0.162				
**Parkinson's (%)**	4 (0.2)	6 (0.9)	5.80 (1.62, 20.74)	0.007	5.12 (1.40, 18.67)	0.013	5.23 (1.44, 19.09)	0.012	5.03 (1.38, 18.31)	0.014
**Diabetes (%)**	230 (8.9)	60 (8.9)	1.00 (0.74, 1.35)	0.994	0.92 (0.68, 1.24)	0.575				
**Education**										
**Primary or less (%)**	351 (13.6)	112 (16.6)	1.00		1.00					
**Secondary (%)**	1617 (62.4)	410 (60.7)	0.79 (0.62, 1.01)	0.056	1.00 (0.78, 1.29)	0.976				
**Degree or higher (%)**	620 (23.9)	152 (22.5)	0.77 (0.58, 1.01)	0.059	0.99 (0.74, 1.32)	0.939				
**Missing (%)**	2 (0.1)	1 (0.1)								
**Smoker**										
**Never (%)**	1367 (52.8)	358 (53.0)	1.00		1.00					

**Former (%)**	954 (36.8)	256 (37.9)	1.02 (0.85, 1.23)	0.803	0.99 (0.82, 1.19)	0.897				
**Current (%)**	267 (10.3)	60 (8.9)	0.86 (0.63, 1.17)	0.327	0.97 (0.71, 1.33)	0.871				
**Missing (%)**	2 (0.1)	1 (0.1)								
**Physical Activity**										
**≥150 minutes per week (%)**	1740 (67.2)	424 (62.8)	1.00		1.00					
**< 150 minutes per week (%)**	828 (32.0)	243 (36.0)	1.20 (1.01, 1.44)	0.042	1.14 (0.95, 1.37)	0.164				
**Cataract**										
**Never (%)**	2122 (81.9)	523 (77.5)	1.00		1.00					
**Present (%)**	305 (11.8)	98 (14.5)	1.29 (1.01, 1.66)	0.042	0.88 (0.67, 1.14)	0.324				
**Surgery (%)**	149 (5.8)	51 (7.6)	1.38 (0.99, 1.94)	0.056	0.83 (0.57, 1.19)	0.309				
**Missing (%)**	14 (0.5)	3 (0.4)								
**Hypertension category**										
**SBP<120 & DBP<80 (%)**	609 (23.5)	133 (19.7)	1.00		1.00		1.00		1.00	
**120≤SBP<130 & DBP<80 (%)**	267 (10.3)	99 (14.7)	1.70 (1.26, 2.29)	<0.001	1.45 (1.07, 1.98)	0.017	1.43 (1.05, 1.95)	0.023	1.42 (1.04, 1.94)	0.025
**130≤SBP<140 Or 80≤DBP< 90 (%)**	1109 (42.8)	290 (43.0)	1.20 (0.95, 1.51)	0.121	1.11 (0.88, 1.41)	0.380	1.11 (0.87, 1.40)	0.406	1.10 (0.87, 1.39)	0.445

**SBP≥140 Or DBP≥90 (%)**	595 (23.0)	149 (22.1)	1.16 (0.89, 1.50)	0.266	1.03 (0.79, 1.35)	0.823	1.02 (0.77, 1.33)	0.908	1.00 (0.76, 1.31)	0.992
**Missing (%)**	10 (0.4)	4 (0.6)								
**Diabetes**										
**No (%)**	2259 (87.2)	585 (86.7)	1.00		1.00					
**Yes (%)**	331 (12.8)	90 (13.3)	1.05 (0.82, 1.35)	0.702	0.90 (0.70, 1.16)	0.417				
**Alcohol intake**										
**0 units/day (%)**	755 (29.2)	232 (34.4)	1.00		1.00					
**0 < units/day < 4 (%)**	1617 (62.4)	401 (59.4)	0.81 (0.67, 0.97)	0.022	0.90 (0.75, 1.09)	0.291				
**≥ 4 units/day (%)**	218 (8.4)	42 (6.2)	0.63 (0.44, 0.90)	0.011	0.76 (0.52, 1.11)	0.153				
**Cognitive**										
**MMSE Score < 24 (%)**	53 (2.0)	11 (1.6)	0.79 (0.41, 1.53)	0.488	0.61 (0.31, 1.20)	0.153				
**MOCA Score < 26 (%)**	1037 (40.0)	303 (44.9)	1.21 (1.02, 1.44)	0.027	1.03 (0.86, 1.23)	0.756				
**MOCA Score missing (%)**	20 (0.8)	2 (0.3)								
**Serum**										
**hsCRP mg/L (SD)**	3.2 (5.3)	3.4 (5)	1.09 (1.00, 1.18)	0.045	1.07 (0.99, 1.16)	0.099	1.08 (0.99, 1.18)	0.065	1.09 (1.00, 1.19)	0.045

**LDL mmol/L (SD)**	3.4 (1.1)	3.3 (1.1)	0.78 (0.61, 1.00)	0.045	1.03 (0.79, 1.33)	0.839				
**HDL mmol/L (SD)**	1.6 (0.4)	1.6 (0.5)	1.29 (0.92, 1.81)	0.134	1.48 (1.02, 2.15)	0.039	1.57 (1.07, 2.31)	0.022	1.58 (1.08, 2.32)	0.019
**Vitamin D ng/mL (SD)**	22 (10.6)	22.6 (10.8)	1.13 (0.94, 1.36)	0.179	1.09 (0.91, 1.31)	0.350				
**Lpa mg/dL (SD)**	20.3 (22.3)	21.8 (22.8)	1.04 (0.95, 1.13)	0.369	1.03 (0.95, 1.13)	0.450				
**Triglyceride mmol/L (SD)**	1.7 (0.9)	1.6 (0.8)	0.84 (0.71, 1.01)	0.065	0.87 (0.72, 1.05)	0.143				
**Creatinine mg/dL (SD)**	0.9 (0.2)	0.9 (0.3)	1.31 (0.88, 1.95)	0.176	0.96 (0.59, 1.54)	0.859				
**ApoA1 g/L (SD)**	1.7 (0.3)	1.7 (0.3)	1.26 (0.70, 2.24)	0.439	1.48 (0.78, 2.80)	0.234				
**ApoB g/L (SD)**	1 (0.3)	1 (0.3)	0.65 (0.47, 0.90)	0.009	0.91 (0.64, 1.27)	0.565				

For continuous variables, the mean and standard deviation is reported. For categorical variables, the number of individuals in each level (category) is given along with the column percentage. Variables that were not included in the regression modelling stage have missing values for all of the Odds Ratio (OR) and associated P value columns. Variables that were considered as candidates for the multiple regression model also have entries in the age and sex adjusted column. Results reported for all regressions are pooled estimates from models fitted to each of the multiple imputed datasets. Therefore, odds ratios are not reported for the “Missing” category of categorical variables because these gaps have been filled in during the multiple imputation procedure. Similarly, missing values in continuous variables have also been filled. SBP=systolic blood pressure, DBP=diastolic blood pressure, MMSE=Mini-mental state examination, MOCA=The Montreal Cognitive Assessment hsCRP=high sensitivity C reactive protein, LDL=low density lipoprotein, HDL= high density lipoprotein, LPA= Lysophosphatidic acid, ApoA1= Apolipoprotein A1 ApoB= Apolipoprotein B. Beckman (0,1)= no signs of retinal aging or druplets $\leq$63 µm

Supplementary Table 5 Risk factor associations for presence vs absence of AMD features on color/*en face* imaging

Participant characteristics	Hyperpigmentation N=111		Drusen (any size) N=1775		Drusen ≥63μm N=833		Drusen ≥125μm N=213		Reticular Drusen N=181	
	OR	P	OR	P	OR	P	OR	P	OR	P
**Age**	1.00 (0.79, 1.28)	0.993	0.95 (0.87, 1.03)	0.197	1.10 (0.98, 1.24)	0.116	1.28 (1.03, 1.59)	0.027	1.06 (0.83, 1.35)	0.660
**Age^2**	1.00 (1.00, 1.00)	0.696	1.00 (1.00, 1.00)	0.091	1.00 (1.00, 1.00)	0.394	1.00 (1.00, 1.00)	0.104	1.00 (1.00, 1.00)	0.835
**Female**	0.66 (0.42, 1.05)	0.080	0.95 (0.81, 1.12)	0.540	1.04 (0.84, 1.28)	0.731	0.77 (0.52, 1.13)	0.181	1.27 (0.83, 1.92)	0.268
**Genetic Risk Score**			1.17 (1.09, 1.27)	<0.001	1.24 (1.13, 1.37)	<0.001	1.52 (1.27, 1.81)	<0.001	1.47 (1.23, 1.75)	<0.001
**Resting heart rate (beats per minute) (SD)**	1.02 (1.00, 1.04)	0.094								
**Spherical Equivalent (Diopters)**			1.07 (1.04, 1.11)	<0.001	1.08 (1.03, 1.13)	<0.001				
Self-report										
**Arthritis**			1.15 (0.99, 1.34)	0.062						
**Parkinson's**	Excluded		2.47 (0.89, 6.82)	0.080	4.18 (1.46, 12.00)	0.007			Excluded	

**Diabetes**									0.51 (0.20, 1.26)	0.143
Physical Activity										
**≥150 minutes per week**					1.00					
**< 150 minutes per week**					1.20 (0.99, 1.45)	0.058				
Cataract										
**Never**									1.00	
**Present**									1.60 (0.98, 2.62)	0.059
**Surgery**									0.83 (0.38, 1.84)	0.651
Hypertension category										
**SBP<120 & DBP<80**			1.00		1.00				1.00	
**120≤SBP<130 & DBP<80**			1.31 (1.03, 1.67)	0.026	1.49 (1.10, 2.03)	0.011			2.42 (1.24, 4.75)	0.010
**130≤SBP<140 Or 80≤DBP< 90**			1.16 (0.97, 1.38)	0.106	1.14 (0.90, 1.46)	0.276			1.65 (0.93, 2.92)	0.086
**SBP≥140 Or DBP≥90**			1.08 (0.88, 1.33)	0.478	1.14 (0.86, 1.52)	0.372			1.60 (0.86, 2.97)	0.138
Alcohol intake										
**0 units/day**	1.00		1.00							

**0 < units/day < 4**	0.60 (0.39, 0.94)	0.026	0.81 (0.70, 0.95)	0.007						
**≥ 4 units/day**	0.66 (0.30, 1.45)	0.299	0.82 (0.62, 1.09)	0.166						
Serum										
**HDL mmol/L**			1.27 (0.92, 1.76)	0.138	3.12 (0.99, 9.84)	0.051	1.35 (0.12, 15.32)	0.807	0.44 (0.04, 4.61)	0.493
**Vitamin D ng/mL**							1.35 (0.95, 1.94)	0.094	1.61 (1.02, 2.52)	0.039
**Triglyceride mmol/L**			0.83 (0.71, 0.97)	0.023	0.94 (0.74, 1.19)	0.596	0.77 (0.49, 1.22)	0.262		
**ApoA1 g/L**					0.34 (0.06, 2.02)	0.234	1.82 (0.04, 89.73)	0.762	9.97 (0.19, 514.12)	0.251
Ocular										
**Normal Choroid 201-400µm**	1.00		1.00		1.00		1.00		1.00	
**Thick Choroid ≥401µm**	1.49 (0.60, 3.69)	0.389	1.79 (1.39, 2.29)	<0.001	1.51 (1.09, 2.10)	0.012	2.15 (1.26, 3.67)	0.005	2.58 (1.35, 4.92)	0.004
**Thin Choroid ≤200µm**	1.78 (1.10, 2.88)	0.019	1.05 (0.88, 1.26)	0.595	0.92 (0.72, 1.17)	0.510	1.04 (0.69, 1.55)	0.866	1.28 (0.83, 1.96)	0.260

Results report the fully adjusted multivariable model for each feature. Results reported for all regressions are pooled estimates from models fitted to each of the multiple imputed datasets. Therefore, odds ratios are not reported for the “Missing” category of categorical variables because these gaps have been filled in during the multiple imputation procedure. Similarly, missing values in continuous variables have also been filled. SBP=systolic blood pressure, DBP=diastolic blood pressure, MMSE=Mini-mental state examination, MOCA=The Montreal Cognitive Assessment hsCRP=high sensitivity C reactive protein,

LDL=low density lipoprotein, HDL= high density lipoprotein, LPA= Lysophosphatidic acid, ApoA1= Apolipoprotein A1 ApoB= Apolipoprotein B, GA=geographic atrophy. Only variables retained in final model are listed in the table, the following were excluded: Waist Hip Ratio, Body Mass Index, self-report cardiovascular disease, self-report chronic lung disease, self-report asthma, self-report cognitive problems, education, smoking, diabetes, cognitive scores, hsCRP, LDL, Lpa, ApoB, creatinine, evidence of epiretinal membrane, evidence of vitreomacular adhesion.

Supplementary Table 6 Eye level risk factor associations with presence vs absence of OCT based AMD features.

Participant characteristics	Classical Drusen N=1688		SDD N=1549		SDD only N=382		Focal Atrophy N=264		CNV N=16	
	OR	P	OR	P	OR	P	OR	P	OR	P
Age	0.97 (0.89, 1.06)	0.486	0.95 (0.86, 1.04)	0.266	0.93 (0.79, 1.11)	0.445	0.89 (0.75, 1.04)	0.139	1.04 (0.53, 2.04)	0.916
Age^2	1.00 (1.00, 1.00)	0.069	1.00 (1.00, 1.00)	0.016	1.00 (1.00, 1.00)	0.187	1.00 (1.00, 1.00)	0.048	1.00 (1.00, 1.01)	0.659
Female	0.99 (0.86, 1.15)	0.910	0.92 (0.79, 1.07)	0.287	1.02 (0.80, 1.29)	0.885	0.82 (0.61, 1.10)	0.190	1.95 (0.59, 6.43)	0.271
Genetic Risk Score	1.33 (1.24, 1.43)	<0.001	1.35 (1.26, 1.44)	<0.001	1.15 (1.02, 1.28)	0.017	1.26 (1.08, 1.46)	0.003	2.07 (1.29, 3.33)	0.003
Spherical Equivalent (Diopters)	1.07 (1.04, 1.11)	<0.001	1.07 (1.03, 1.10)	<0.001						
**Self-report**										
Chronic lung disease							1.96 (1.05, 3.68)	0.034	Excluded	
Cancer							0.50 (0.26, 0.96)	0.037	Excluded	
Parkinson's	3.34 (1.10, 10.15)	0.033							Excluded	
**Education**										
Primary or less	1.00		1.00				1.00		1.00	

Secondary	0.90 (0.73, 1.10)	0.294								
Degree or higher	0.89 (0.70, 1.12)	0.311								
**Smoker**										
Never	1.00		1.00				1.00		1.00	
Former							1.38 (1.01, 1.88)	0.041	2.47 (0.76, 8.09)	0.133
Current							1.21 (0.72, 2.04)	0.458	3.19 (0.32, 32.26)	0.323
**Hypertension Category**										
SBP<120 & DBP<80	1.00		1.00		1.00		1.00		1.00	
120≤SBP<130 & DBP<80	1.29 (1.00, 1.65)	0.047	1.28 (0.99, 1.65)	0.058	1.41 (0.89, 2.24)	0.141				
130≤SBP<140 Or 80≤DBP< 90	1.08 (0.89, 1.30)	0.427	1.06 (0.88, 1.29)	0.517	1.37 (0.98, 1.92)	0.060				
SBP≥140 Or DBP≥90	1.11 (0.90, 1.37)	0.344	1.14 (0.91, 1.41)	0.247	1.34 (0.93, 1.93)	0.118				
**Cognitive**										
MOCA Score < 26			0.84 (0.73, 0.98)	0.024						
**Serum**										

hsCRP mg/L	1.06 (0.99, 1.13)	0.105					1.11 (0.97, 1.28)	0.138	1.72 (1.01, 2.94)	0.047
Lpa mg/dL					0.90 (0.80, 1.01)	0.077			1.77 (0.96, 3.24)	0.068
Triglyceride mmol/L	0.83 (0.71, 0.96)	0.014								
ApoA1 g/L			1.53 (0.93, 2.50)	0.090						
**Ocular factors**										
Evidence of epiretinal membrane	0.83 (0.66, 1.05)	0.122	0.76 (0.60, 0.95)	0.014	0.62 (0.39, 0.96)	0.032	1.36 (0.87, 2.12)	0.179	2.83 (0.80, 10.10)	0.106
Evidence of vitreomacular adhesion					1.21 (0.92, 1.59)	0.176	0.72 (0.53, 0.99)	0.040		
Normal Choroid 201-400µm	1.00		1.00		1.00		1.00		1.00	
Thick Choroid ≥401µm	1.84 (1.42, 2.37)	<0.001	1.34 (1.01, 1.77)	0.041	1.55 (0.90, 2.66)	0.109			5.00 (0.71, 35.09)	0.104
Thin Choroid ≤200µm	0.89 (0.74, 1.07)	0.210	1.09 (0.91, 1.31)	0.361	1.67 (1.26, 2.22)	<0.001			1.93 (0.60, 6.16)	0.265

For continuous variables, the mean and standard deviation is reported. For categorical variables, the number of individuals in each level (category) is given along with the column percentage. Results reported are from the fully adjusted regression models. Results reported for all regressions are pooled estimates from models fitted to each of the multiple imputed datasets. Therefore, odds ratios are not reported for the "Missing" category of categorical variables because these gaps have been filled in during the multiple imputation procedure. Similarly, missing values in continuous variables have also been filled. SBP=systolic blood pressure, DBP=diastolic blood pressure, MMSE=Mini-mental state examination, MOCA=The Montreal Cognitive Assessment hsCRP=high sensitivity C reactive protein, LDL=low density lipoprotein, HDL= high density lipoprotein, LPA= Lysophosphatidic acid, ApoA1= Apolipoprotein

A1 ApoB= Apolipoprotein B, SDD=subretinal drusenoid deposits, CNV=choroidal neovascular membrane. Only variables retained in final model are listed in the table, the following were excluded: Waist hip ratio, Body Mass Index, Resting heart rate, self-report cardiovascular disease, self-report asthma, sel-report artritis, self-report cognitive problems, self-report arthritis, self-report diabetes, cataract, diabetes, alcohol intake, MMSE Score <24, LDL, HDL, Vitamin D, creatinine, Apo B.
